# Microstructure, local viscoelasticity and cell culture suitability of 3D hybrid HA/collagen scaffolds

**DOI:** 10.1371/journal.pone.0207397

**Published:** 2018-12-19

**Authors:** Johanna Roether, Sarah Bertels, Claude Oelschlaeger, Martin Bastmeyer, Norbert Willenbacher

**Affiliations:** 1 Institute for Mechanical Process Engineering and Mechanics, Applied Mechanics Group, Karlsruhe Institute of Technology (KIT), Karlsruhe, Germany; 2 Department of Cell- and Neurobiology, Zoological Institute, Karlsruhe Institute of Technology (KIT), Karlsruhe, Germany; Illinois Institute of Technology, UNITED STATES

## Abstract

As mechanical properties of cell culture substrates matter, methods for mechanical characterization of scaffolds on a relevant length scale are required. We used multiple particle tracking microrheology to close the gap between elasticity determined from bulk measurements and elastic properties sensed by cells. Structure and elasticity of macroporous, three-dimensional cryogel scaffolds from mixtures of hyaluronic acid (HA) and collagen (Coll) were characterized. Both one-component gels formed homogeneous networks, whereas hybrid gels were heterogeneous in terms of elasticity. Most strikingly, local elastic moduli were significantly lower than bulk moduli presumably due to non-equilibrium chain conformations between crosslinks. This was more pronounced in Coll and hybrid gels than in pure HA gels. Local elastic moduli were similar for all gels, irrespective of their different swelling ratio and bulk moduli. Fibroblast cell culture proved the biocompatibility of all investigated compositions. Coll containing gels enabled cell migration, adhesion and proliferation inside the gels.

## 1 Introduction

Scaffolds for successful tissue engineering must be biodegradable and biocompatible, with an open, macroporous three-dimensional architecture and should have appropriate mechanical properties closely mimicking those of the natural extra cellular matrix (ECM) [1].

Mechanical properties play a fundamental role in resistance and stability of the gels but also alter cell migration, adhesion, proliferation and metabolism [2–9]. In the past, mechanical properties of hydrogels were generally characterized using bulk rheological measurements [3,4,6,7,10–12], as well as uniaxial compression tests [13–17]. These latter assess the Young’s modulus E which characterizes bulk elasticity of an entire sample on a macroscopic scale. Different moduli are connected to different tissue applications, from soft mucosa with E ~ kPa to hard bone tissues with E ~ GPa. However, cell behavior is significantly influenced by the elasticity of the direct microenvironment [18], which may not be well characterized by the bulk elastic modulus, particularly, when the gel composition, i.e. the polymer concentration or cross-link density is spatially heterogeneous and/or the gel includes pores. Cells probe the elasticity of their surrounding in the range of up to five times their length (reviewed in [19]) by actively pulling fibers they are adhered to. Whether the displacement of fibers or the corresponding force of the material is sensed, is subject of current discussion [8]. According to the fiber pulling theory, the local properties of pore walls in water filled macroporous scaffolds are more relevant, than bulk elasticity. But pore wall/ material thickness should be taken into account, as the force a cell has to apply for buckling of a strut depends on the geometry and elasticity of this object [19]. Some studies exist in the literature where local viscoelastic properties of the surfaces of cell culture substrates were investigated by means of atomic force microscopy (AFM) based nano/micro indentation and cell behavior was said to be affected by the determined matrix elasticity [18,20–23]. Here it is important to keep in mind, that cells do not necessarily sense the scaffold surface and that apparent elasticity of soft materials depends on the used measurement method [24]. However, matrix stiffness caused changes in cell morphology, cell differentiation, cell spreading and proliferation [25–28]. Besides that, growing fibroblast cells themselves affect ECM mechanical properties during remodeling, depending on initial scaffold properties [19,29,30]. In an iterative process, those altered properties of the remodeled matrix feedback to cell growth. Daviran et al. [31] investigated the degradation of non-porous poly(ethylene glycol)-peptide hydrogels by enzymes secreted from encapsulated cells using a microrheology method and Kuboki et al. [22] showed that the secretion of Coll by seeded cells in addition to the Coll already present increases the matrix stiffness. Additionally, cells increase Coll network density by contraction during remodeling [32]. To our knowledge, for porous hydrogels, only one attempt [33] was made to characterize matrix local viscoelastic properties. Indentation experiments were employed in this case, the new insight, however, was limited due to various drawbacks. A first limitation of this experimental approach is the difficulty to identify the point of zero force. A second one is the softness of the material. Cryogels are considered as soft materials with a Young´s modulus E < 1 MPa whereas indentation techniques are more adapted for stiff materials with E > 1 GPa. In conclusion, the study of soft porous hydrated materials still poses various challenges demanding innovative characterization techniques providing accurate information about local viscoelastic properties of soft hydrogels.

In this study we used the cryogelation method [34] to fabricate hybrid macroporous scaffolds from hyaluronic acid (HA) and collagen (Coll) mixtures using ethylene glycol diglycidyl ether (EGDE) as chemical crosslinker and we employed multiple particle tracking (MPT) microrheology to determine the local viscoelastic properties of these soft gels. The goal was to produce cryogels with controlled pore size, wall thickness, and viscoelastic properties for application in cell culture. We wanted to understand how sample composition and local viscoelastic properties of the matrix affect cell behavior. For that, in the first part of the study, we investigated the influence of HA and Coll concentrations on gel swelling capacity, pore size and matrix thickness, as well as macro and micro-mechanical properties. The second part was dedicated to the cultivation of mouse dermal fibroblast cells, incorporated into the macroporous scaffolds. Cell viability, proliferation and morphology were characterized. Finally, the in-vitro biodegradability of the scaffolds was investigated.

## 2 Materials and methods

### 2.1 Preparation of HA/Coll cryogels

Macroporous gels were prepared using the cryogelation technique as described in Oelschlaeger et al. [35] Briefly, sodium hyaluronate (HA), M_w_ = 2.2 Mio Da, Contipro, CZ) was dissolved in a 0.25 M sodium hydroxide (Carl Roth, Germany) aqueous solution under constant stirring for 20 min, and this mixture was maintained for 16 h at 4 °C until complete dissolution. Coll solutions were prepared following the manufacturers recommendations, by dissolution of Coll (Collagen I, fibrous powder from bovine tendon, AdvancedBioMatrix, USA) in 5 mM hydrochloric acid (Carl Roth, Germany). To ensure appropriate dissolution of Coll and exclude phase separation, the solution had to be mixed for 18 h with magnetic stirrer under ambient temperature, leading to a highly viscous liquid. Rheological properties of solutions of HA, Coll and mixtures are shown as S1 Fig in the supporting information. Hybrid HA/Coll solutions were made of the two solutions by blending them under stirring. After adjusting pH of blended solutions to 13.2 ± 0.2 with concentrated NaOH, 0.7 wt% of crosslinker EGDE (Sigma, USA) were added. By stirring for 30 min, uniform distribution of EGDE was ensured. The solutions were poured into cylindrical PTFE molds (diameter 10 mm, height 3 mm) and tightly sealed. Thereafter molds were placed into an ethylene glycol (Carl Roth, Germany) bath and stored at -20 or -80°C for 6 days. After freezing, gels were allowed to warm up to room temperature for at least 2 h before performing experiments. The repetition of this preparation routine led to five independent batches that were used for characterization. All cylindrical cryogel specimen were immersed in bi-distilled water and all experiments were performed in wet state.

The swelling ratio was determined by measuring the ratio of the mass of the gel in the swollen (wet) and un-swollen (dry) state. To ensure sufficient statistical significance, weight was averaged over 10 different of 5 independent batches. In particular, dry gels were weighed directly after fabrication (m_dry_) and again after being immersed in water for 4 h (m_wet_, measured in triplicate for each specimen, to take into account the influence of remaining surface water). Swelling ratio is mainly controlled by the interconnectivity of the pores and the water up-taking capacity of the used polymers.

### 2.2 Scaffold pore size and shape, network thickness and topology characterizations

The overall scaffold architecture was investigated firstly using laser scanning microscopy (LSM, LSM 510, Carl Zeiss, Germany). For visualization of the pore walls and for MPT measurements (see 0), green fluorescent polystyrene particles (diameter 0.19 μm, Bangs Laboratories) were added to the solution before freezing so that particles remained in the polymer phase during gelation and were finally entrapped exclusively in the pore walls. Secondly, swollen specimen were immersed in Rhodamine B solutions for 3 days and after excessive washing in water investigated by a confocal laser scanning microscope (CLSM, TCS SP8, Leica Microsystems, Germany), combined with a 20x multi-immersion objective. A comparison of gels stained with both methods showed a high degree of co-localization, so particles are considered to be distributed all over the polymer network (data not shown).

### 2.3 Multiple particle tracking based optical microrheology

MPT was developed as a microrheological tool that allows for the characterization of microstructural and micromechanical properties of many materials [36] (and references therein). Studying cryogels, we have used this technique to characterize local viscoelastic properties of the matrix and viscous properties of the pore filling liquid. The fluid mechanics of microrheology and especially the principles and applications have been described in detail [37,38]. The underlying idea of MPT is to monitor the Brownian motion of inert colloidal probe particles embedded in a material and thereby obtain quantitative information about the rheological properties of the surrounding fluid. This technique was introduced in the mid-1990s when Mason and Weitz proposed a quantitative relation between the tracer mean square displacement (MSD) 〈*Δr*^2^(τ)〉 as a function of lag time τ and the macroscopic complex shear modulus G*(ω) as a function of the frequency *ω* [39]. The Laplace transform of the particle MSD $\langle \Delta {\tilde{r}}^{2}(\text{i}\omega )\rangle$ is related to the complex modulus G* of the sample via a generalized Stokes–Einstein equation (GSE, general form for 3D, see Eq 1) [40]:

$$G^{*}\left(\omega \right)=\frac{k_{B}T}{\pi ai\omega \langle \Delta {\tilde{r}}^{2}\left(\text{i}\omega \right)\rangle }=G^{\prime }\left(\omega \right)+iG^{\prime \prime }\left(\omega \right)$$ (1)

*a* stands for the radius of the embedded beads, *k*_*B*_ for the Boltzmann constant and *T* for the temperature. This GSE relation is valid only under the assumption that the material surrounding the sphere can be treated as an isotropic and homogeneous continuum, i.e. that the particle size is larger than the structural length scales of the probed material. For the cryogels investigated here, the mesh size calculated from macrorheological measurements ranged from 4–17 nm (see section 4.2.2), which is much smaller than the size of the particles we used (diameter 200 nm). Furthermore, probe particle and fluid inertia can be neglected, Reynolds number *Re* and Stokes number *Stk* both are well below 1.

For 2D tracking of beads suspended in an ideal elastic material, Eq 1 reduces to Eq 2 [41] including a prefactor of 2/3 for the numbers of dimensions [42]:

$$G_{0}=\frac{2k_{B}T}{3\pi a\langle \Delta r^{2}(\tau )\rangle }$$ (2)

Where *G*_*0*_ is the shear modulus of the material independent of ω. All cryogels investigated here, behave like elastic solids, as confirmed by the time-independence of the MSD at times <0.3s, independent of the matrix composition. Therefore, we used Eq 2 to determine local matrix elasticity, *G*_*0*_ hereinafter referred to as *G*_0,*MPT*_.

Our setup is based on an inverted fluorescence microscope (Axio Observer D1, Carl Zeiss, Germany) equipped with a Fluar 100x objective (numerical aperture 1.3, 100x magnification, oil immersion lens, Carl Zeiss). We tracked the Brownian motion of green fluorescent polystyrene microspheres of 0.19 μm diameter (see 3.2) in two dimensions. In isotropic materials, no additional information is obtained from 3D tracking and by reducing the measurement to 2D, the performance of the system is enhanced. For performing MPT measurements exclusively in the matrix, particles were added to the polymer solutions before freezing. In order to exclude protein absorption on the particle surface, which would affect the measured diffusivity, we compared measurements with native polystyrene (PS) particles to PS particles functionalized with Polyethylene glycol (donated by Xabier Murgia, Department of drug delivery, Helmholtz Institute for Pharmaceutical Research Saarland). Particle diffusion was similar for both particle types confirming that the effect of adhering protein or HA on the particle surface is negligible. To perform MPT experiments in the pore liquid, tracer particles of 0.5 μm diameter were locally added to pores of polymerized swollen samples using a syringe.

Images of these fluorescent beads were recorded via a sCMOS camera Zyla X (Andor Technology, Ireland: 21.8 mm diagonal sensor size, 2160 × 2160 square pixels). The displacements of particle centers were monitored in a 127 × 127 μm field of view at a rate of 50 frames/sec. This latter value is the maximum rate of image capture that our camera can achieve, so that the temporal resolution at short timescales is limited to 0.02s. Movies of the fluctuating microspheres were analyzed using a custom MPT routine, including the software Image Processing System (Visiometrics iPS) and a self-written Matlab code [36], based on the widely used Crocker and Grier tracking algorithm [43]. We examined the distribution of displacements, known as the Van Hove correlation function [44] and calculated the non-Gaussian parameter α according to Eq 3 [45].

$$\alpha =\frac{\langle {\text{x}}^{4}(\tau )\rangle }{3\langle {\text{x}}^{2}{(\tau )\rangle }^{2}}-1$$ (3)

This parameter describes the derivation of the MSD values from a Gaussian distribution expected for a homogeneous uniform sample and characterizes the heterogeneity of the sample on a 0.1–1 μm length scale.

### 2.4 Bulk mechanical properties

#### 2.4.1 Compression test

Uniaxial unconfined compression tests were performed at room temperature using the commercial tensile testing machine Texture Analyzer TA.XTplus (Stable Micro System, UK) equipped with a 5 kg load cell. Tests were performed on cylindrically shaped gels (diameter << plate size) of different height and diameter, depending on the degree of swelling. Samples were compressed up to 80% strain at a compression speed of 0.5 mm/s. The strain ε was calculated as the ratio of the change in length during compression Δl and initial height of the sample l_0_. Using this, Young’s modulus E was determined as the slope of the initially linear stress-strain curve in the strain region < 5%, (see S2 and S3 Figs).

#### 2.4.2 Oscillatory shear

In shear, the gels were characterized through their storage modulus G’ and loss modulus G” as a function of frequency. Measurements were performed using a rotational rheometer (Physica MCR501, Anton Paar) with a plate-plate geometry (diameter 8 mm). Gap height was adjusted between 1 and 2 mm depending on the height of the swollen samples to obtain a normal force of 0.15 ± 0.05 N. For all compositions, frequency sweeps were performed in the linear regime at a stress amplitude of τ = 0.5 Pa, covering the frequency range of 0.1 to 10 rad/s.

### 2.5 Degradation kinetics

Disk-shaped gels (initial diameter: 10 mm, height: 3 mm) of different composition were placed in beakers filled with water (ten times the initial weight of the individual specimen). Individual specimens were used for compression tests or MPT measurements after different periods of time, from one day to 500 days.

### 2.6 In vitro assessment of biocompatibility

The thawed gels were swollen in water and washed with PBS (Phosphate Buffered Saline. PAN Biotech). Afterwards they were placed in 12-well plates and immersed in DMEM (Dulbeco’s Modified Eagles Medium, PAN Biotech), supplemented with 10% FCS (Fetal Calves Serum, PAN Biotech). Cells were passaged according to a routine protocol and cultivated under standard conditions (37°C, 5% CO_2_, 90% rel. humidity).

NIH-3T3 fibroblast cells were detached using 0.25% trypsin/EDTA (ethylenediaminetetraacetic acid) in PBS and suspended in supplemented medium. Cells were seeded onto the gels at a density of 75 000 cells per gel (235 mm^3^). Initially, they were allowed to settle without additional medium for 30 min at 37°C. Later, 2 mL medium were added and the cells were cultivated for 8 days. Medium was exchanged every second day. For each independent experiment, gels from a newly synthesized batch were used.

Live/dead assay was performed after 1, 3 and 8 days by addition of 0.5 μl Calcein (Thermo Fischer Scientific, 4 mM in DMSO (dimethyl sulfoxide)) and 2 μl Ethidium homodimer (Thermo Fischer Scientific, 2 mM in DMSO/water) directly into the 2 ml nutrient medium in each well. Resulting concentrations were 0.25 μl Calcein/ml medium and 1μl Ethidium homodimer/ml medium. After incubation for 15 min, at least 5 LSM images were recorded during 15 min. In all images, living and dead cells were counted and the count was averaged for all images.

For characterization of cell morphology, the gels were first fixed with 4% para-formaldehyde (30 min, 20°C), then washed twice with PBS and permeabilized with Triton X-100 in PBS for 20 min.

Cell division events were investigated after 1, 3, and 8 days in culture by EdU-labeling. The labeling procedure was performed with the Click-it^™^ EdU Alexa Fluor^™^ 594 Imaging kit according to the manufacturer’s instructions. Scaffolds were washed with 1% BSA (bovine serum albumin in PBS) after fixation and permeabilization. Anti-Actin and DAPI staining was performed subsequently. Therefore, scaffolds were immersed in primary antibody solution (anti-actin, Sigma Aldrich, A2066 1:200 in 1% BSA) for 6h. After washing with PBS twice, scaffolds were placed in secondary antibody solution (goat-anti-rabbit Alexa Fluor^™^ 647, Jackson Immuno Research, 1:250 and DAPI, Carl Roth, 1:1000 in 1% BSA) over night at -4°C. Prior to imaging, scaffolds were washed with PBS twice. At least 5 images per scaffold were taken and ratio of number of EdU positive cells to total cell count was averaged.

All experiments were done in triplicate. For each independent run, new gels were produced.

## 3 Results and discussion

### 3.1 Structural properties of HA, Coll and hybrid cryogels

**Fig 1:**
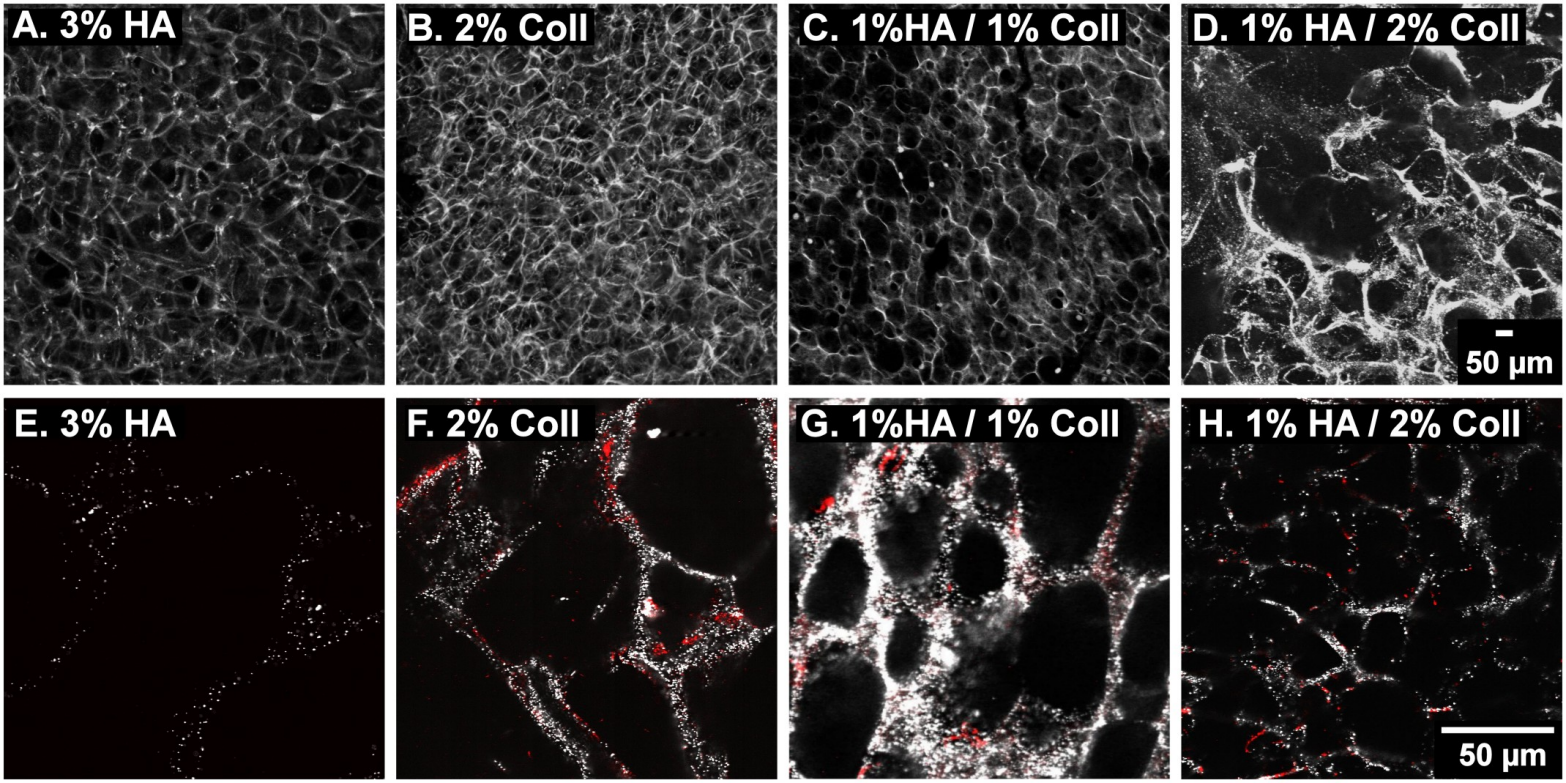
Morphology of swollen cryogels. LSM images of fluorescent tracer particles localized in the gel network. 3D stack (A-D) and corresponding 2D image, 40x magnification, with anti-Coll staining shown in red (E- H).

Pore size and shape of cell-free gels of different compositions were qualitatively determined from LSM images. For visualization of the network structure, the fluorescence signal of embedded tracer particles was recorded. Fig 1 shows images of hydrated cryogels with a total polymer concentration of 2–3 wt% composed of 3% HA (Fig 1A and 1E), 2% Coll (Fig 1B and 1F) as well as a mixtures of HA and Coll (Fig 1C, 1D, 1G and 1H).

The 3%HA, 2%Coll and 1%HA/1%Coll gels all exhibit a fairly roundish pore shape in the swollen state, but pore size varies. Pure HA gels have larger pores ~100–120 μm in diameter (Fig 1A and 1E) compared to ~50 μm pores in pure Coll (Fig 1B and 1F) and ~50 μm pores in 1% HA/1% Coll gels (Fig 1C and 1G). The matrix thickness and variation of pore size are similar in these gels. However, the gel consisting of 1%HA/2%Coll (Fig 1D and 1H), shows a strong local variation in pore size. In some areas, pores were narrower than 20 μm, elsewhere big cracks disturbed the continuous network.

To get more information about the Coll distribution in the hybrid matrix, we stained Coll I (shown in red) using polyclonal α-Coll I (rabbit) primary antibody in combination with Cy3-labeled (goat) secondary antibody (Fig 1E–1H).

As expected, no Coll is present in pure HA gel (Fig 1E), while in pure Coll (Fig 1F), interconnected Coll fibers are visible along all pore wall structures. In both hybrid scaffolds (Fig 1G and 1H), the Coll network is interrupted by unstained sections, were only tracer particles are visible indicating a non-homogeneous Coll distribution in the matrix.

### 3.2 Swelling and bulk mechanical properties

#### 3.2.1 Swelling

**Fig 2:**
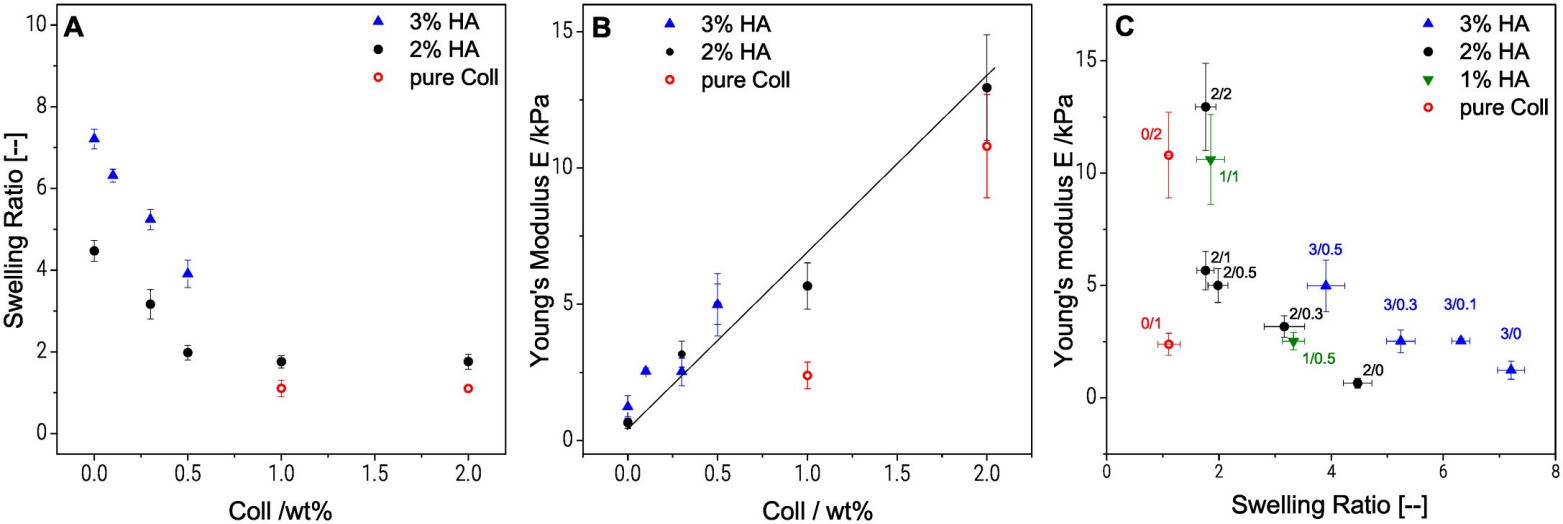
Influence of swelling behavior on bulk elasticity of cryogels. Swelling ratio (A) and Young’s modulus measured in uniaxial compression (B) over Coll concentration, and influence of degree of swelling on Young’s modulus for samples with different HA/Coll content in wt% (C). Young´s modulus values represents average and standard deviation obtained from at least 25 specimens of each composition.

As mentioned previously, one of the distinctive properties of macroporous HA gels is to swell instantaneously when immersed in water or in a standard cell culture medium [46]. The excessive swelling of HA gels seems to be related to the ability of glycosaminoglycans with their large number of hydrophilic groups and flexible three-dimensional structure, to bind lots of water [47]. Swelling capacity of Coll free gels increased from 4.35 ± 0.37 for 2%HA gels to 7.21 ± 0.24 for 3%HA gels (see Fig 2A). As the total amount of crosslinker was kept constant (0.7 wt %) this increased swelling capacity is related to the lower crosslinker/polymer ratio, i.e. decreased crosslink density. An increase in swelling ratio with lower crosslinker/polymer ratio at constant HA content was observed before [34].

Furthermore, Fig 2A shows the effect of the Coll concentration on swelling ratio in water. For both HA concentrations, we observed a linear decrease of swelling capacity when the Coll content was increased to 0.5 wt %. For the gel with 2 wt % HA, the swelling ratio decreased from 4.35 ± 0.37 to 1.98 ± 0.18 when the Coll concentration was increased from 0 to 0.5 wt % and levelled off at a constant swelling ratio upon further increase of Coll concentration. This decrease in swelling capacity, also seen elsewhere [48,49], can be related to a reduction of the HA/Coll network flexibility due to the rigidity of Coll fibers. These latter serve as a cage, hindering the expansion of the flexible HA polymer coils.

Finally, for cryogels composed only of Coll, swelling ratio was close to one, i.e. these gels essentially do not swell. Similar observations have been reported for natural cartilage, where Coll can also take-up water, but due to the dense rigid fibrous structure, swelling is limited [50]. The Coll network is apparently so stiff that it cannot expand and swelling is not possible.

#### 3.2.2 Cryogel bulk elasticity in uniaxial compression and shear

Fig 2B shows that Young’s modulus E in the wet state, increases almost linearly from 0.5 to 13 kPa, when the Coll content in 2 wt % HA gels is increased from 0 to 2 wt %. A reinforcement of the matrix with increasing Coll concentration is expected in the dry state due to the formation of strong Coll fibers randomly distributed within the network. These fibers made of polypeptide chains are known to form stable helical structures in alkaline solutions at room temperature [5]. However, in the wet state, the effect of swelling is dominating. Less swollen, especially Coll containing gels show higher elastic moduli compared to highly swollen gels (see Fig 2C). As seen in Fig 2B, the Young’s modulus of 2%HA (E = 0.65 ± 0.21 kPa) is similar to the one of 3%HA (E = 1.1 ± 0.5 kPa), though the swelling ratio is substantially higher for 3%HA gels (7.2 ± 0.2, compared to 4.5 ± 0.3 for 2% HA). This is consistent keeping in mind that the crosslinker concentration is the same in both cases but more polymer between network junctions is available to preserve the shape of the swollen gel including 3%HA. The lower crosslinker to polymer ratio in the 3%HA gel leads to an increased swelling ratio for the 3%HA gel, but the Young’s modulus is similar as for 2%HA, because due to the increased total polymer content, the density of entanglements among polymer chains is higher than in 2%HA gel and this contributes to the elasticity of the gel, too. The contribution of entanglements to the modulus is on the same order of magnitude as that of the crosslinks [35].

Finally, we observed that the Young´s modulus of the cryogel composed of only Coll was higher by a factor of 15 compared to the one obtained for HA (both 2 wt %), while swelling is four times lower compared to pure HA gels. Even at a concentration of 1%Coll the modulus is five times higher than that of 2%HA gel. This confirms the high compressive strength of Coll networks and the strong influence of the degree of swelling.

**Fig 3:**
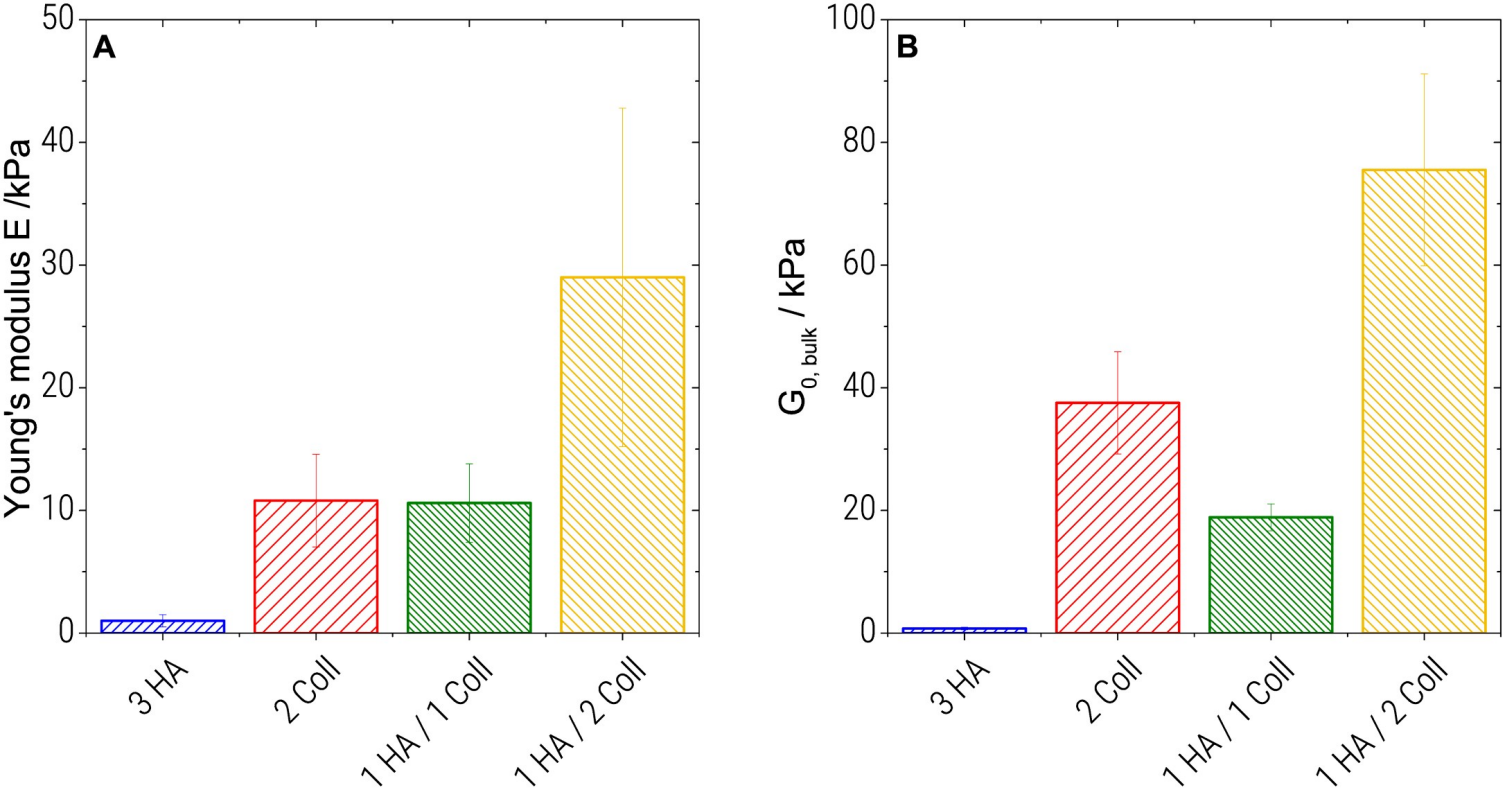
Bulk mechanics of cryogels. Young’s modulus determined in uniaxial compression (A) and shear modulus obtained from small amplitude oscillatory shear (B).

As seen if Fig 3A, the pure 3%HA gel (E = 1.1 ± 0.5 kPa) is weaker in uniaxial compression, compared to pure 2%Coll gels (E = 11 ± 4 kPa) or hybrids (11 ± 3 kPa for 1%HA/1%Coll and 29 ± 14 kPa for 1%HA/ 2%Coll). Bulk elasticity values of 1%HA/2%Coll scatter strongly which might be related to the heterogeneous structure seen in Fig 1. This latter gel exhibits the highest modulus despite its higher degree of swelling compared to the pure 2%Coll sample. However, an increased modulus for heterogeneous structures was observed in various other polymer systems before, e.g. acrylic thickeners [36,51] or methacrylate copolymers [52]. Uniaxial compression of a macroscopic, swollen specimen, is not only determined by the matrix elasticity. Besides the degree of swelling (see 4.2.1), structural properties, such as pore size, pore shape, pore interconnectivity and wall thickness may also affect Young’s modulus.

In oscillatory shear, samples of all compositions show frequency independent elastic moduli and G’ dominates over G” in the frequency range from 0.1 to 100 rad/s (see S1 Fig). This is considered typical gel-like behavior. Corresponding shear modulus data G_0,bulk_ (average of G’ values obtained in the probed frequency range) are shown in Fig 3B. As in uniaxial compression, the highly swollen pure HA samples appear weaker (G_0,bulk_ = 0.8 ± 0.2 kPa) than Coll containing samples. Pure Coll (G_0_,_bulk_ = 38 ± 8 kPa) exhibits a higher modulus than the 1%HA/1%Coll mixture (G_0_,_bulk_ = 19 ± 2 kPa). The highest shear modulus was found for the 1%HA/2%Coll mixture (G_0_,_bulk_ = 76 ± 16 kPa).

In conclusion, the bulk shear and compression moduli are on the same order of magnitude and a similar ranking within the series of investigated samples is found. However, the G_0_,_bulk_ values are somewhat higher than corresponding Young’s modulus values. This is in contrast to the E = 3*G_0_ relationship expected for uniform, isotropic bodies and may be attributed to the heterogeneous porous structure of the gels investigated here.

Finally, from above *G*_0,*Bulk*_ values, we can directly determine the mesh size *ξ*_*Bulk*_ of the scaffold network according to the classical theory of rubber elasticity assuming thermal equilibrium (Eq 4) [53]:

$$G_{0,Bulk}=\frac{k_{B}T}{\xi_{MPT}^{3}}$$ (4)

In all cases, *ξ*_*Bulk*_ varied between 17 ± 2 nm (pure HA) and 4 ± 0.2 nm (1% HA / 2% Coll). These values are significantly smaller than the diameter of the embedded tracer particles (diameter = 200 nm) we used for MPT measurements. Consequently, this result confirms the validity of Eq 2, namely that the material can be treated as a continuum on the length scale that is sensed by the particles.

### 3.3 Local viscoelastic properties from multiple particle tracking microrheology

We employed multiple particle tracking (MPT) microrheology for characterization of local viscoelastic properties of the HA/Coll matrix as well as viscous properties of the pores. Independent of the sample composition, we observed purely diffusive motion of the tracer particles dispersed in the pores, i.e. the microenvironment surrounding the particles responded like a viscous liquid (data not shown). The obtained viscosity was close to that of water and in all cases these pore filling solutions were homogeneous as indicated by the value of the non-Gaussian parameter α ≅ 0 (see Eq 3).

**Fig 4:**
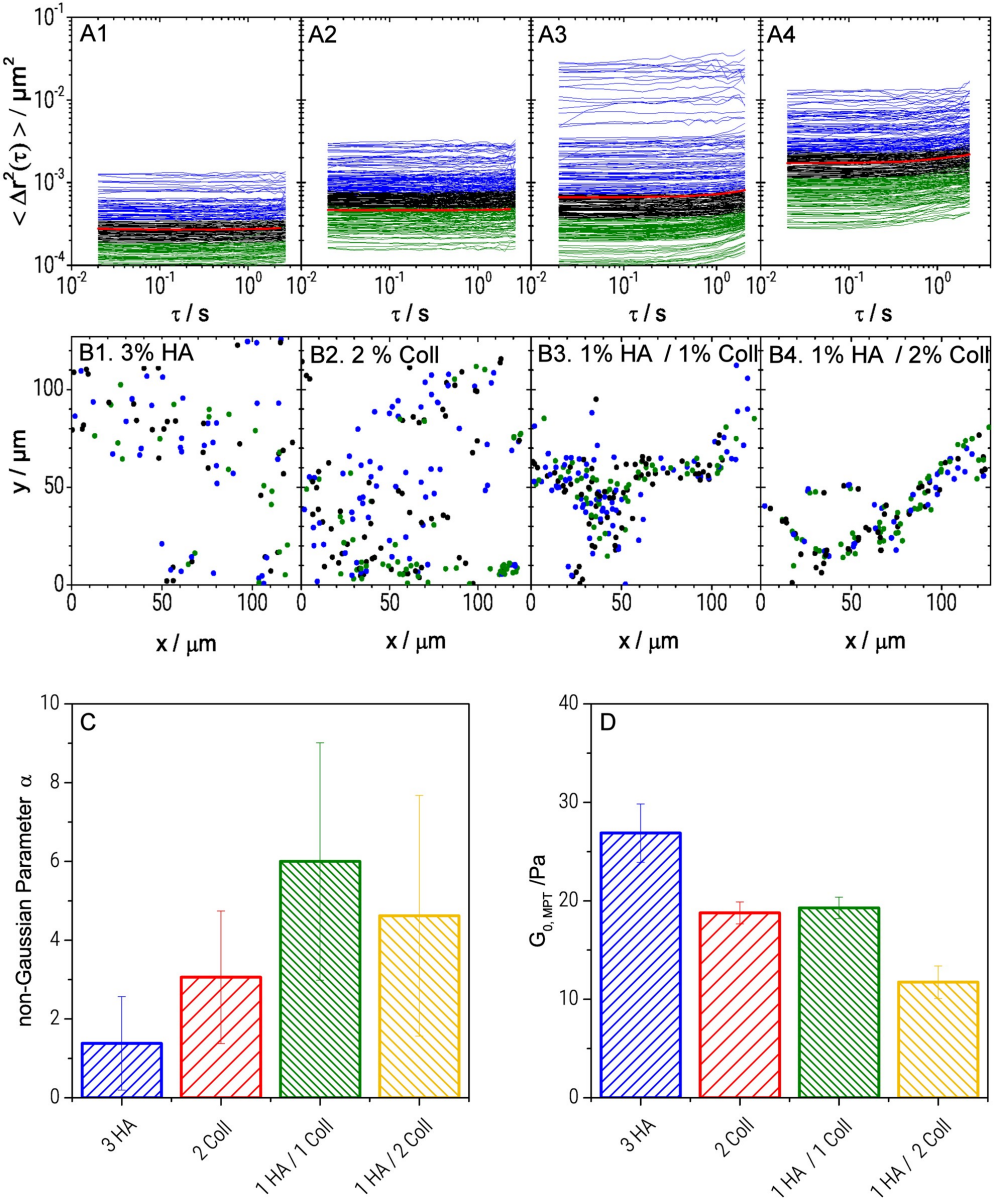
Local characterization of HA and Coll scaffolds. MSD plots from MPT measurements (A), corresponding trajectories (B), (color code: blue refers to highly mobile particles (highest third of MSD values), green corresponds to the almost immobile ones (lowest third of MSD values) and black is used for the middle third of MSD values), local heterogeneity characterized in terms of the non-Gaussian parameter α (τ = 0.1s) calculated for the ensemble of MSDs according to Eq 3 (C): α values were obtained averaging data from 4 videos (~200 particles /frame) that were recorded at different localizations of at least two samples of each batch. The given error bars show the standard deviation of corresponding evaluated material parameters, and local storage modulus (D).

The motion of particles dispersed in the cryogel network (Fig 4A) was significantly different from diffusion in the pores. For both, single component HA (Fig 4A1) and single component Coll (Fig 4A2) scaffolds, MSD curves were time independent (dΔr^2^/dτ ≈ 0) throughout all probed time scales and showed an average MSD (red curve) value of 2.7 ± 0.2x10^-4^ μm^2^ and 4.6 ± 0.3x10^-4^ μm^2^, respectively. This indicates that particles were highly constrained by their surrounding which is consistent with an elastic trapping of tracer particles in a gel-like network. In contrast, HA/Coll mixtures (Fig 4A3 and 4A4) showed viscoelastic behavior, as the corresponding MSD plots exhibited an upward curvature at long lag times (τ > 1 sec), indicating slow viscous diffusion of the beads corresponding to a transition into the terminal flow regime.

Furthermore, the analysis of the MSD distribution provides information about the degree of heterogeneity of the matrix and the local variation of viscoelastic properties with microscale resolution. For the pure HA gel (Fig 4A1), the range of displacement at a given lag time was quite narrow. At τ = 0.1 sec, MSDs varied within one order of magnitude, from 10^−4^ to 10^−3^ μm^2^. For pure Coll gel (Fig 4A2), the range of displacement was slightly broader, MSDs covered a range of almost 1.5 decades from 10^−4^ to 3x10^-3^ μm^2^. Corresponding values of the non-Gaussian parameter α were α = 1.4 ± 1.2 and α = 3.1 ± 1.7 for pure HA and Coll gel, respectively (see Fig 4C). This result indicates that both, pure HA and pure Coll network, were essentially homogeneous.

Higher α-values were found for the hybrid gels (α = 6.0 ± 3.0 for 1%HA/1%Coll and α = 4.6 ± 3.1 for 1%HA/2%Coll gels). Despite the large uncertainty in determination of the α values due to a strong variation of MSD distributions obtained in different regions of a sample, it is obvious that the matrix heterogeneity was more pronounced for these hybrid gels with a variation of the absolute MSD values within 2.5 orders of magnitude (Fig 4A3 and 4A4). Distribution and length scale of heterogeneities were mapped by plotting all particle positions in the plane of observation (Fig 4B) and coloring each individual trajectory according to the MSD’s absolute value. Blue color corresponds to highly mobile particles (highest third of MSD values), green color corresponds to the almost immobile ones (lowest third of MSD values) and black is used the middle third of MSD values.

Independent of the matrix composition, mobile and immobile particles were homogeneously distributed all over the sample without any pattern and the length scale of heterogeneity was shorter than the mean distance between particles. Finally, we determined the local elastic plateau modulus *G*_0,*MPT*_ from the time- independent average MSD (τ<0.3s) using Eq 2. We found *G*_0,*MPT*_ values ranging from 26.9 ± 3.0 Pa for pure HA to 11.7 ± 1.7 Pa for 1% HA/ 2% Coll. (Fig 4D) leading to apparent mesh size *ξ*_*MPT*_ values varying between 53 ± 4 nm (pure HA) and 70 ± 7 nm (1% HA / 2% Coll). Note, calculation of *G*_0,*MPT*_ is less affected by the strong variation of MSDs than the determination of the heterogeneity parameter α as visible from the smaller relative standard deviations for the former quantity.

Most strikingly, the local elastic moduli *G*_0,*MPT*_ were much lower (and naturally mesh sizes calculated from these were higher) than the corresponding bulk shear moduli *G*_0,*Bulk*_ shown in Fig 3 consistent with results previously reported for pure HA gels [34]. This might be due to a pronounced contribution of stretched out of equilibrium chain segments between network junctions as observed earlier for keratin networks [54] or due to densely crosslinked areas not accessible for the tracer particles and thus not contributing to *G*_0,*MPT*_ but showing up in the bulk modulus. Exposing Collagen to an acidic environment during our sample preparation can lead to a loss of telopeptides [55] resulting in a decrease of the number of ligand binding sites relevant for the molecular packing structure of the Collagen. This might explain the existence of such densely packed molecular structures. This latter hypothesis, however is not consistent with the uniform distribution of tracer particles visible in Fig 1 and in [34]. The ratio *G*_0,*Bulk*_/*G*_0,*MPT*_ is about 30 times larger for the 2%Coll gel (*G*_0,*Bulk*_/*G*_0,*MPT*_ ≈ 2000) than for the 3%HA gel (*G*_0,*Bulk*_/*G*_0,*MPT*_ ≈ 65) suggesting that out of equilibrium network strands formed during cryogelation are more important for the stiffer Coll chains than for the highly flexible HA polymers. This effect is even more pronounced for the mixed gels for which *G*_0,*Bulk*_/*G*_0,*MPT*_ values of 4000 (1% HA/2% Coll) and 700 (1%HA/1%Coll) are found, similar findings were reported for other biomaterials before but not specifically addressed [56]. Table 1 summarizes the numerical values of the different mechanical parameters obtained for all investigated gel compositions.

**Table 1:** Numerical values of E, G_0,Bulk_, G_0,MPT_ and α as for different gel compositions. Values are shown as mean and standard deviation.

	3% HA	2% Coll	1% HA / 1% Coll	1% HA / 2% Coll
E / kPa	1 ± 0.5	11 ± 4	11 ± 3	29 ± 14
G_0,Bulk_ / kPa	0.7 ± 0.2	37 ± 8	19 ± 2	75 ± 16
G_0,MPT_ / Pa	27 ± 3	19 ± 1	19 ± 1	12 ± 2
α / [-]	1.4 ± 1.2	3.1 ± 1.7	6.0 ± 3.0	4.6 ± 3.1

### 3.4 Degradability

**Fig 5:**
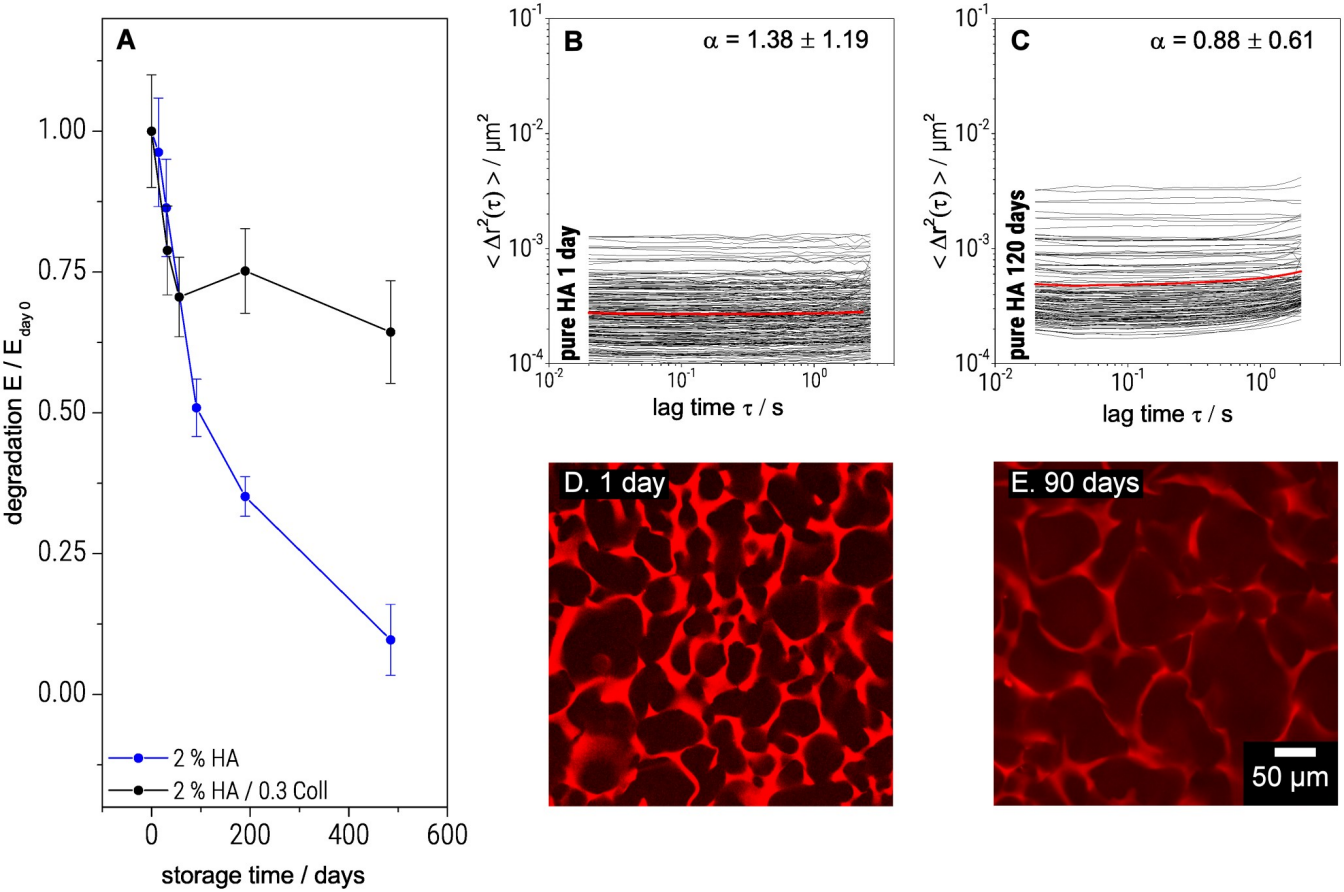
Degradation of cryogels. Decrease in Young’s modulus over storage time(A), Changes in MSD plots after 1 day (B) and after 120 days (C) and corresponding LSM images of pure HA gels stained with Rhodamine B after 1 day (D) and 90 days (E). The resulting Young’s moduli were averaged over three to five samples at each specific point in time. The shown error bars represent the corresponding standard deviation.

Scaffolds used for culturing cells should initially support and stabilize the growing tissue. Then they should gradually degrade when the regenerated tissue starts to develop its own mechanical integrity and strength. Information about scaffold degradation kinetics is thus important for a targeted tissue engineering. Fig 5A shows the degradation of one component HA and hybrid HA/Coll scaffolds in water at 20°C, expressed in terms of Young’s modulus. HA scaffolds degraded almost linearly in time, and after 500 days, E was only 10% of the initial value. For the hybrid scaffold, including additionally 0.3% Coll, the modulus decreases during the first 100 days until it reaches 70% of its initial value. Afterwards it remains constant for up to 500 days, indicating that Coll fibers are less sensitive to degradation by water, than HA polymer networks.

Degradation was also investigated using MPT. Fig 5B and 5C show the variation of MSDs as a function of lag time τ for a 2%HA scaffold after one day and 120 days immersed in water, respectively. The local plateau modulus G_0,MPT_, deduced from the average MSD curve decreased by 60% of the initial value from 29 ± 8 Pa to 10 ± 2 after 120 days. Similar experiments on a mixed gel (2% HA / 0.3% Coll) revealed a decrease by only 20% of the initial value from 6.8 ± 3 to 5.4 ± 2 Pa. The strong decrease in G_0,MPT_, i.e. increase in particle mobility, in the pure HA network is due to the degradation of HA chains and a corresponding decrease in crosslink density. In the presence of Coll, this degradation was much weaker. Apparently, Coll fibers are less sensitive to chain scission. Additionally for aged gels, a slight upward curvature of the MSD plots at high lag times was observed (Fig 5C and see also 4.3). This viscoelastic response indicates defects in the degraded network. Besides the decrease in gel elasticity, degradation resulted in an increase of the pore size, as shown in Fig 5D and 5E. The pores of pure HA scaffolds grew from 100 to 300 μm within 90 days in water. In contrast, for the mixed gel (2% HA / 0.3% Coll) no increase in pore size was observed and bulk elasticity was not further affected after 60 days of storage.

### 3.5 Biocompatibility of HA/Coll cryogels

All tested compositions appeared suitable for cell culture. During cultivation of 3T3 fibroblasts for 8 days, viability and cell division rate were both on a high level.

**Fig 6:**
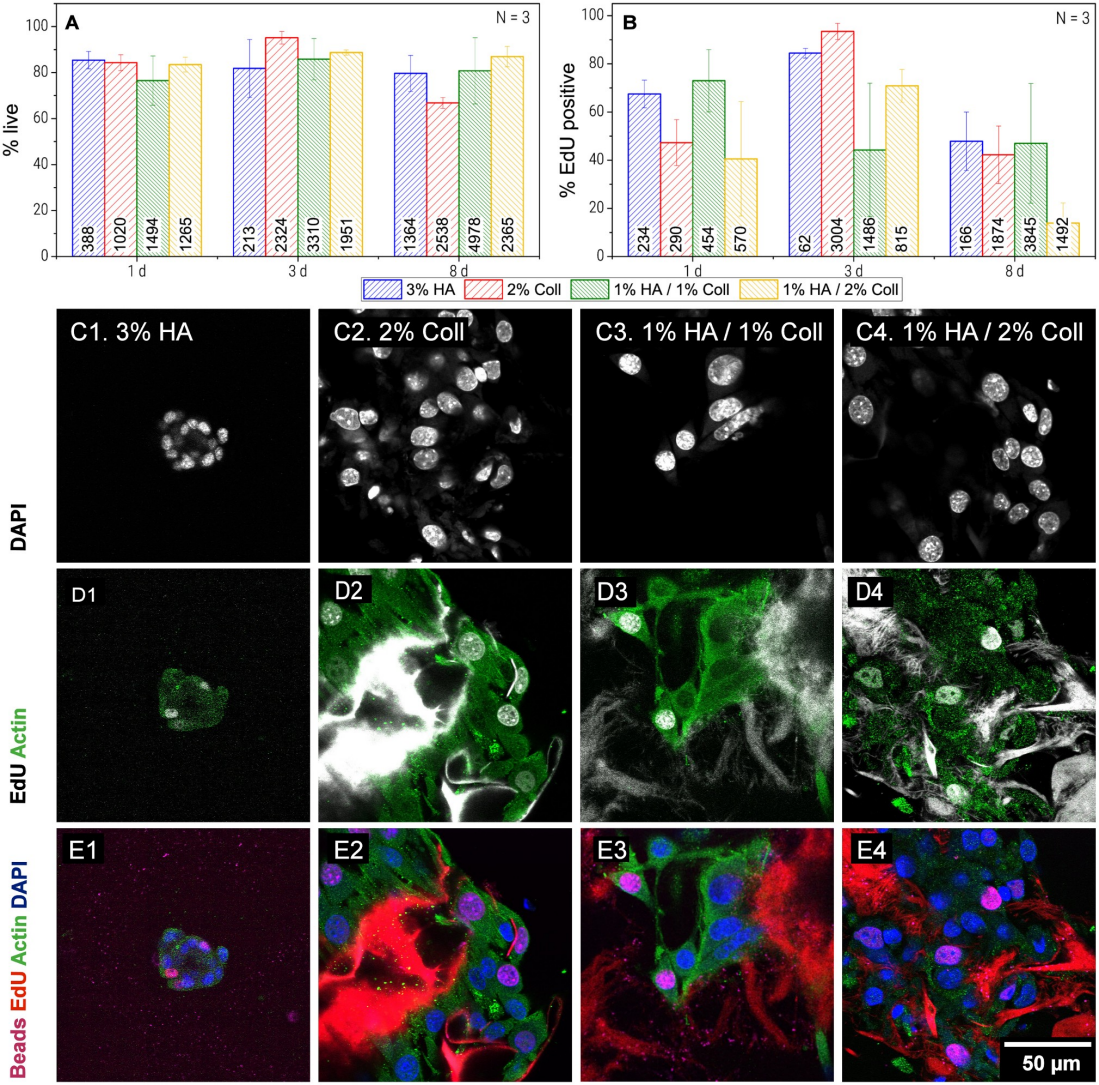
Biocompatibility of cryogel scaffolds. Survival rate (A), proliferation rate (B), presented as mean of N = 3 independent experiments with standard deviation. Morphology of 3T3 cells cultivated in cryogels for 8d: Cell cores stained with DAPI (C), Actin (green) and EdU positive cells and EdU stained network (gray). Coll I network showed bright fluorescence signal, when stained by the EdU assay used here. In HA only light background fluorescence was observed. (D) and merge of all channels (E).

As shown in Fig 6A, viability investigated after 8 days was slightly higher in HA containing gels (79 ± 8% for pure HA gel, 80 ± 14% for 1%HA1%Coll and 87 ± 4% for 1%HA2%Coll) compared to pure Coll (66 ± 2%). However, in 1%HA/2%Coll, only few cell division events were observed (14 ± 8% EdU positive cells at day 8, see Fig 6B). This might be related to insufficient nutrient supply in the denser areas of these heterogeneous gels (see 4.1) and this was further supported by the fact that cells settle preferentially close to cracks, because here flow of nutrient media is facilitated. Additionally, as shown in Fig 1, this gel possessed comparably thick pore walls. As cells are said to probe their environment by sensing the force needed to deform structures [19], they might prefer the more homogeneously distributed pores and thinner pore walls in 1%HA/1%Coll mixtures or pure Coll gels. In fact, in pure Coll gel and 1%HA/1%Coll mixtures, investigation of cell morphology (Fig 6C, 6D and 6E) showed that cells spread and adhered well forming confluent layers all over the network structures. Whether the broader pore size distribution (see Fig 1) or the increase in heterogeneity of local matrix elasticity (see Fig 4E) was responsible for this change, needs to be further investigated.

Interestingly, after 3 days of cell culture, pure Coll (95 ± 3% live cells, 93 ± 3% cell division rate) was superior in terms of cell division and viability, compared to all other samples.

In pure HA viability and cell division rate (48 ± 12% EdU positive cells) were surprisingly high after 8 days. However, overall cell numbers were low, since active cell migration into the pure HA gel was impeded by the lack of adhesive protein patterns. But obviously, appropriate cultivation conditions were available for the few cells that were transported into the gels passively. Those were, as expected because of lacking adhesive structures, not able to spread and stayed in aggregates of up to 50 cells (see Fig 6C1, 6D1 and 6E1). However, the variation in micro- and macro-elasticity of the gels investigated here is too small to explain differences in cell behavior by aspects of mechanotransduction. On macro scale, as well as on microscale, moduli of all tested compositions are in the same range. But the ratio of macro to micro modulus is significantly different for pure HA gels and Coll containing gels (see 4.3). In order to clarify whether cell behavior is affected by this elasticity ratio, i.e. cells can sense the free energy of their environment or the number of stretched out of equilibrium chain segments between network junctions, additional tests with separate variation of composition and elasticity ratio will be necessary.

To conclude, our scaffolds have a high degree of biocompatibility. Coll is, as already known, necessary for cell adhesion and 1% Coll/1% HA is the most attractive gel for cell growth. In terms of long-time proliferation, it might be even more favorable than pure Coll, which is currently used commonly.

## 4 Conclusions

We investigated the influence of Coll concentration on material properties and cell culture suitability of HA based cryogel scaffolds. Firstly, we were able to show that increasing Coll concentrations reduces swelling of porous HA gels, while pure Coll gels do not swell at all, though they are porous as well. The elastic properties of the gels are mainly depending on the degree of swelling, which makes pure Coll gels to appear stiffer compared to pure HA gels with the same overall polymer content.

All different types of scaffolds were proven to be suitable for long term culture of fibroblasts and the introduction of Coll improves mimicry of natural ECM and enables cells to adhere to the scaffolds.

In 1%HA/2%Coll gels, cells were able to migrate deep into the scaffold and viability as well as proliferation were both satisfying during 8 days of cell culture.

Biodegradability of cryogels is drastically reduced when small fractions of Coll are incorporated into HA gels (in vitro, immersed in water). We are able to tailor the mechanical, chemical and degradation properties of macroporous, biobased, biofunctional cryogel scaffolds and their function over persistence time in a wide range by varying the amounts of HA and Coll, crosslinker concentration and process parameters.

On microscale, HA and Coll single component gels are both relatively homogeneous, whereas HA/Coll mixtures showed heterogeneity in network elasticity and pore shape. The local elasticities measured by MPT were significantly lower, compared to bulk elastic moduli, which might be related to a contribution of stretched out of equilibrium chain segments between network junctions. Whether cell survival and proliferation are affected by the enthalpic energy density of the surrounding gel mainly contributing to the bulk modulus or by the local thermally excited network response has to be addressed in future research.

However, with the results presented in this study, we were able to show, that MPT can help for accurate microscale characterization of complex biomaterials. In order to study the material properties that are sensed by cells, geometrical and micromechanical characterization has to be brought together.

## Supporting information

S1 Fig Bulk rheological measurements of precursor solutions and cryogels. Frequency sweeps were measured with CP20 (solutions) and PP08 (gels) in the linear viscoelastic regime.
(TIF)

S2 Fig Exemplary force vs time plots of multiple strain cycles. (TIF)

S3 Fig Exemplary stress-strain plots resulting from one-time uniaxial compression of cryogel cylinders. Young’s moduli were calculated from data on the strain region < 5%, which is highlighted in grey.
(TIF)
